# YAP Transcriptional Activity Dictates Cell Response to TNF *In Vitro*

**DOI:** 10.3389/fimmu.2022.856247

**Published:** 2022-03-23

**Authors:** Robin Caire, Elisa Dalix, Marwa Chafchafi, Mireille Thomas, Marie-Thérèse Linossier, Myriam Normand, Alain Guignandon, Laurence Vico, Hubert Marotte

**Affiliations:** ^1^ INSERM, U1059-SAINBIOSE, Université de Lyon, Saint-Etienne, France; ^2^ Department of Rheumatology, Hôpital Nord, University Hospital Saint-Etienne, Saint-Etienne, France

**Keywords:** YAP, TNF, Rho-GTPases, inflammation, fibrosis

## Abstract

YAP/TAZ are transcription co-factors recently described responsive to pro-inflammatory cytokines and involved in inflammatory-related disorders. However, the role of tumor necrosis factor (TNF), a major pro-inflammatory cytokine, on YAP signaling is not well understood and controversial. Here, we observe *in vitro*, using wild type and YAP knockout HEK293 cells, that TNF triggers YAP nuclear translocation and transcriptional activity, thus being dependent on Rho family of GTPases. In response to TNF, YAP transcriptional activity orientates cell fate toward survival. Transcriptional analysis with Nanostring technology reveals that YAP modulates TNF-induced increase in fibro-inflammatory pathways such as NF-κB, inflammasomes, cytokines or chemokines signaling and pro-fibrotic pathways involving TGF-β and extracellular matrix remodeling. Therefore, in response to TNF, YAP acts as a sustainer of the inflammatory response and as a molecular link between inflammation and fibrotic processes. This work identifies that YAP is critical to drive several biological effects of TNF which are involved in cancer and inflammatory disorders.

## Introduction

Yes associated protein (YAP) and WW domain-containing transcription regulator protein 1 (WWTR1, classically referred as TAZ) are transcriptional co-activators that were originally described important for organ growth control (1). The main transcriptional partner of YAP/TAZ are TEA domain transcription factors (TEADs 1 to 4), which promote survival and migrative abilities in cells (1, 2). Because of these abilities, YAP/TAZ are well known for their role in promoting tumorigenesis (3, 4). The inputs controlling YAP translocation and transcriptional activity are numerous. First, a kinase cascade belonging to the Hippo pathway led to YAP/TAZ phosphorylation (including serine 127 phosphorylation of YAP) and retention in the cytoplasm or degradation in the proteasome preventing its role of co-transcription factor (5, 6). Second, conditions leading to cellular tension activate the YAP/TAZ pathway (7). This activation depends on integrins and Rho family of GTPases (RhoA, RhoB, and RhoC) signaling allowing the tension of actin cytoskeleton and the formation of actin stress fibers (7–9). YAP/TAZ are also controlled by actin severing and capping proteins promoting their cytoplasmic localization in a context of low mechanical stress (10). Other inputs controlling YAP/TAZ activation have been identified such as G-protein coupled receptors (GPCRs) (11) and Wnt (12) signaling pathways. Bodies of evidence also indicate that inflammation and pro-inflammatory cytokines are YAP/TAZ modulators (13).

**Graphical Abstract:**
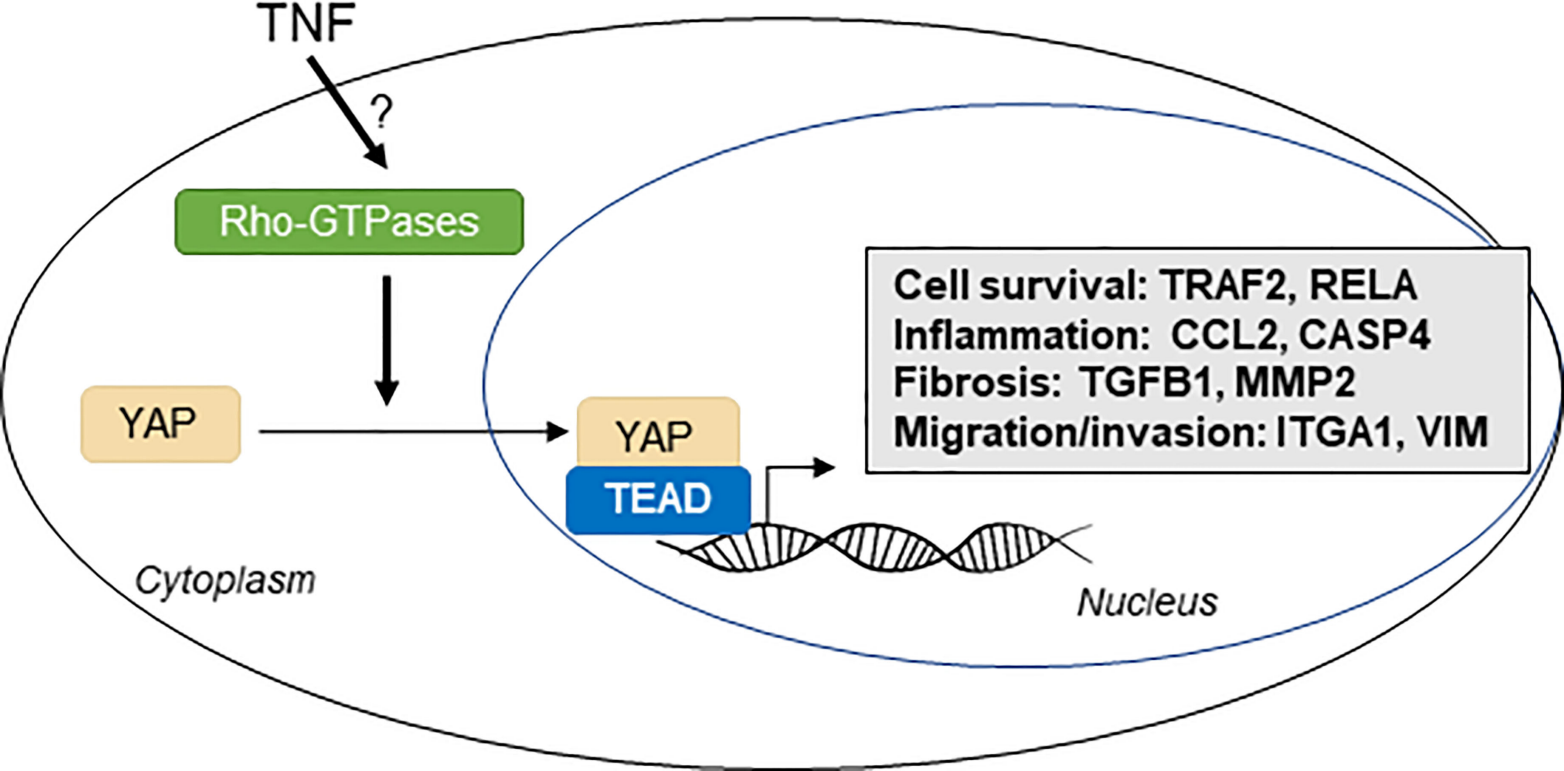


Inflammation is a conserved process that has physiological roles for pathogen defense and tissue repair after injury (14). YAP is critical for regenerative processes in an important number of tissues (8, 13), but how inflammation modulates this process is not completely understood. The link between inflammation and YAP is still unclear since the activation of YAP by pro-inflammatory cytokines remains controversial. Among pro-inflammatory cytokines, tumor necrosis factor α (TNF) is considered as a master pro-inflammatory regulator and exerts pleiotropic biologic effects. TNF is produced by many cell types and acts principally through TNF receptor 1 (TNFR1), which is expressed in virtually any cell types. TNF signaling has a dual role on cell survival. It activates NF-κB and JNK-MAPK signaling thus promoting anti-apoptotic and pro-inflammatory response, but it can also lead to cell death by apoptosis induction (15, 16). This dual regulation depends on molecular complexes, such as TNF receptor associated factor (TRAF) proteins and especially TRAF2, which plays an important role by orientating TNF response through cell survival (16, 17). Thus, TNF is known to promote tumor progression by enhancing the proliferation of tumor cells (18). TNF is a strong promotor of inflammation and is involved in pro-inflammatory-related diseases (19, 20). TNF signaling increases the expression of an important range of cytokines and chemokines such as IL-6, IL-8 (also known as CXCL8) (20). TNF also enhances pro-inflammatory response by increasing intercellular adhesion molecule‐1 (ICAM‐1) and vascular cell adhesion molecule‐1 (VCAM‐1) expressions and the release of chemokines such as CCL2, thus enhancing the recruitment of immune cells (20). TNF activates several other inflammatory pathways such as inflammasome and interferon signaling (21). Besides its role in inflammation, TNF also promotes focal adhesion kinase pathway and the re-organization of the actin cytoskeleton, with the formation of actin stress fibers (22–24). TNF also activates Rho family of GTPases (25). These mechanisms are important for cell migrative and invasive abilities. TNF induces the expression of matrix metalloproteinases (MMPs), which helps for the degradation of ECM components and therefore promotes cell invasion and metastasis (26, 27). For these reasons, TNF promotes metastasis in cancer cells and invasive abilities of resident cells of the joint during rheumatoid arthritis (18, 19). In another hand, TNF promotes the expression of pro-fibrotic genes such as connective tissue growth factor (CTGF) and TGF-β (a master regulator of fibrosis) and is therefore critically implicated in pro-fibrotic processes (28–30). Altogether, a better understanding on how TNF acts at the cellular level to exerts these effects is useful for the understanding of both physiological and pathological processes and, therefore, for the development of novel therapeutic strategies.

Interestingly, CTGF is also known to be a specific target of YAP/TEAD transcriptional activity (31), and its transcript level measurement is classically used to assess YAP/TEAD transcriptional activity. It seems also that TNF effect on actin cytoskeleton could promote YAP activation since YAP control by F-actin is critical. Moreover, YAP was shown to be partially activated by Rho family of GTPases signaling in response to TNF in endothelial cells (32). TNF activates YAP in breast cancer cells (33), endothelial cells (32) and synovial cells (34). On the opposite, 1 and 6 h of TNF administration lead to YAP transcriptional activity decrease both in chondrocytes and in HEK293 cell (35, 36). HEK293 cell is a common cell lineage used to investigate fundamental YAP/TAZ biology (12). Thus, the effect of TNF on YAP signaling remains controversial. Moreover, specific regulation differences between YAP or TAZ upon TNF administration were not investigated. On the other hand, YAP promotes the expression of several pro-inflammatory cytokines, chemokines and NF-κB pathway genes (13, 37). However, YAP specific knockdown in endothelial cells promotes systemic inflammation in mice and YAP knockout in HEK293 cells lead to increase NF-κB activity (35, 38). Thus, it is critical to clearly determine the effect of pro-inflammatory cytokines, in particular TNF, on YAP/TAZ *in vitro*; and to decipher how in response, YAP/TAZ orientate cell response.

Here, we investigate at different timing the effect of TNF on YAP/TAZ signaling in HEK293 cells and assess, if, in response YAP/TAZ are important for TNF effect on cell phenotype. We hypothesize that TNF induces YAP/TEAD transcriptional activity that in turn mediates several well-known effects of TNF. We found that long-term TNF administration (from 24 to 48 h) was needed to increase YAP/TEAD transcriptional activity through Rho family of GTPases. We also demonstrate that YAP is critical for mediating TNF effect on cell fate, by orienting cell response toward survival and for mediating an inflammatory transcriptional profile induced by TNF. We also discover that YAP transcriptional activity drives TNF effect on pro-fibrotic genes expression and actin cytoskeleton dynamic. Our results highlight that YAP is a new important effector of TNF signaling *in vitro*.

## Results

### TNF Increases YAP/TEAD Transcriptional Activity

**Figure 1:**
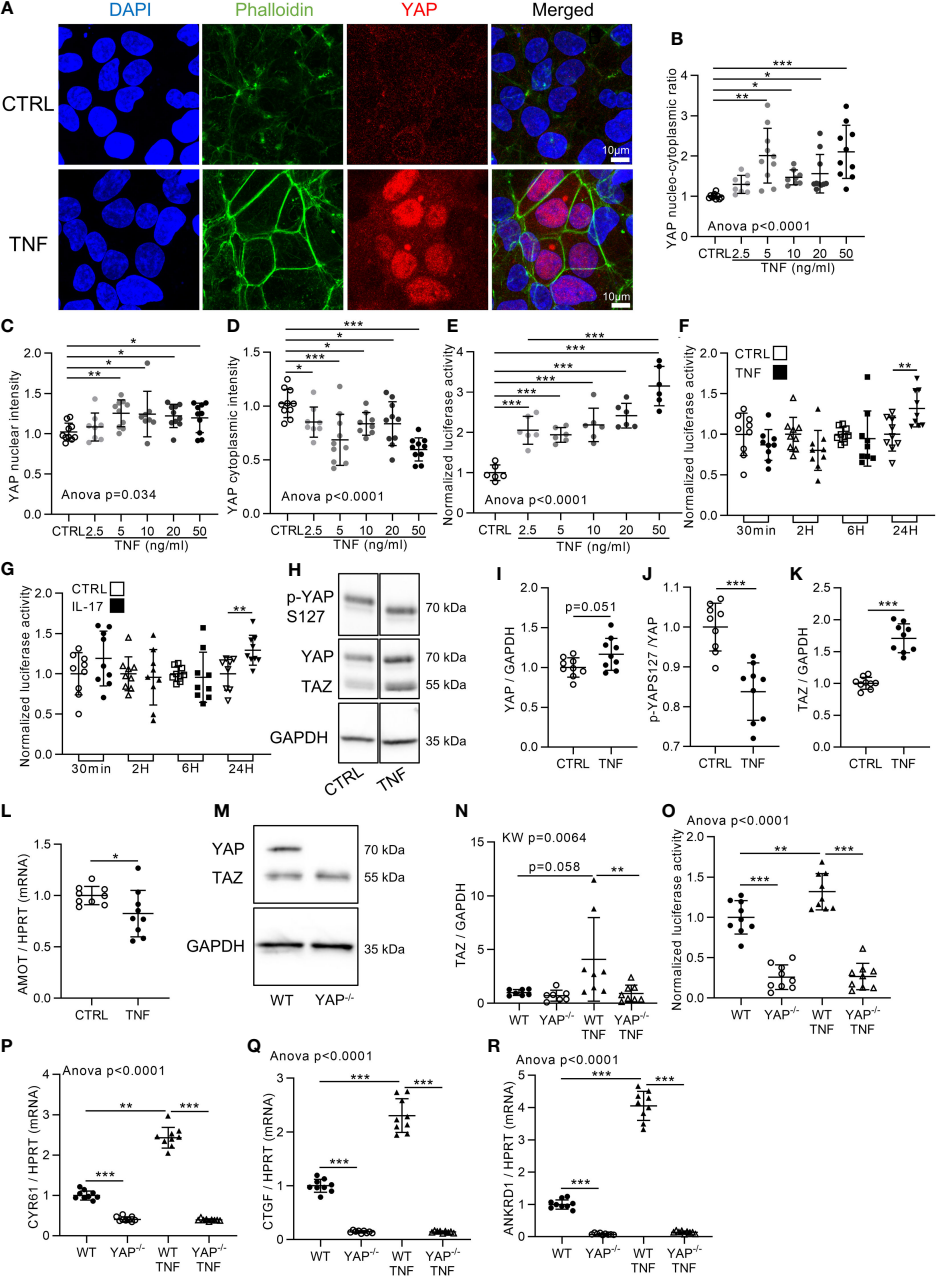
YAP transcriptional activity is increased by TNF and IL-17. HEK293 were seeded at 100,000 cells/cm^2^ on fibronectin coated culture plate. Approximately 24 h after seeding cells were treated with TNF at 10 ng/ml for 48 h [or 24 h for **(F)**, **(G)** and **(O)**]. **(A)** Representative airyscan confocal z-stack max intensity images of YAP (immunofluorescence (IF) technique, red), phalloidin (actin, green), DAPI (nucleus, blue), and merged images (luminosity and contrast were enhanced identically for each image for clarity purpose). **(B–D)** Corresponding IF quantification, with the nucleo-cytoplasmic ratio of YAP labeling **(B)**, YAP mean nuclear intensity **(C)** and YAP mean cytoplasmic intensity **(D)**. **(E–G)** Luciferase reporter assay of TEAD transcription factor activity upon different TNF concentrations at 24 h of treatment **(E)** or at different timings with 10 ng/ml of TNF **(F)** or 50 ng/ml of IL-17 **(G)** as indicated. **(H–K)** Representative western blot of YAP phosphorylation on serin 127 (YAPS127), YAP/TAZ and GAPDH for control or TNF treated cells **(H)** with their respective quantifications as indicated **(I–K)**. **(L)** RT-qPCR result for AMOT, results were normalized to HPRT expression. **(M, N)** Representative western blot of YAP/TAZ and GAPDH for WT and YAP^−/−^ cells **(M)** with respective TAZ quantification **(N)**. **(O)** Luciferase reporter assay of TEAD transcription factor activity in WT and YAP^−/−^ control or TNF treated cells. **(P–R)** RT-qPCR results for CYR61 **(P)**, CTGF **(Q)**, and ANKRD1 **(R)**, results were normalized to HPRT expression. Results are representative of three independent experiments with 2 to 4 biological replicates for each experiment (n = 6 to 10 per group) with T-test or one-way ANOVA test and FDR corrected for multiple comparisons *post hoc* tests performed between conditions: *p < 0.05; **p < 0.01; ***p < 0.001. Data are expressed as fold change vs. control and presented as individual values with mean ± SD.

Cell confluency is an important input regulating YAP activity (7). Accordingly, HEK293 cells were used at two cell densities: high density (HD; 100,000 cells/cm^2^) and low density (LD; 10,000 cells/cm^2^). As expected, YAP was localized in the cytoplasm at HD, whereas it was in the nucleus at LD (**Figure 1A** and **Figure S1A**). To investigate inflammation effect on YAP/TAZ activity *in vitro*, HEK293 cells were stimulated with TNF for 48 h at both densities, allowing us to investigate a potential negative or positive impact on YAP nuclear localization. TNF treatment did not change YAP localization at LD, showing that TNF did not decrease YAP nuclear localization (**Figures S1A–D**). However, at HD, TNF increased YAP nuclear intensity and decreased YAP cytoplasmic intensity resulting in an increase of YAP nucleo-cytoplasmic ratio at 48 h from 5 to 50 ng/ml (**Figures 1A–D**). Furthermore, TEAD transcriptional activity was also strongly increased by TNF treatment from 2.5 to 50 ng/ml (**Figure 1E**). Thus, TNF strongly promoted YAP nuclear translocation and TEAD transcriptional activity at HD. Accordingly, all further investigations were performed at HD. Previous results indicated that TEAD transcriptional activity was decreased after 6 h of TNF treatment in HEK293 cells ectopically transfected with YAP expression plasmid (35). Thus, TEAD activity was assessed in our model by TNF treatment at different timings by focusing only on YAP endogenous level in HEK293 cells. No significant changes in TEAD transcriptional activity were detected until 24 h of treatment, where TEAD activity was increased (**Figure 1F**). Similar results were observed with interleukin-17 (IL-17), another pro-inflammatory cytokine (**Figure 1G**). In western blot, YAP total protein trended to increase (**Figures 1H–I**). YAP phosphorylation at S127 (controlled by Hippo pathway) was reduced by TNF treatment (**Figures 1H-J** **)** suggesting that TNF inhibits the Hippo pathway, therefore reducing YAP retention in the cytoplasm. Total TAZ protein level clearly increased upon TNF treatment (**Figures 1H-K** **)**. Furthermore, angiomotin (AMOT) expression, describes to promote YAP retention in the cytoplasm (39), was reduced by TNF treatment (**Figure 1L**). Then, to confirm that TEAD transcriptional activity was mediated by YAP/TAZ and to better discriminate the individual respective role of YAP or TAZ, YAP knockdown by CRISPR-Cas9 technology was performed on HEK293 cells. To begin, the lack of YAP protein in YAP^−/−^ cells was confirmed while TAZ protein level was unchanged (**Figures 1M, N**). In wild type (WT) HEK, TNF increased TEAD activity and YAP/TAZ target genes expression: ankyrin repeat domain-containing protein 1 (ANKRD1), cysteine-rich angiogenic inducer 61 (CYR61), and CTGF (**Figures 1O–R**) which are specific YAP/TAZ target genes (7, 40). In YAP^−/−^ cells, TEAD activity and YAP target genes expression were strongly downregulated compared to WT cells. Furthermore, TNF treatment failed to induce TEAD activity and YAP/TAZ target genes expression in YAP^−/−^ cells showing that YAP KO alone was sufficient to prevent TEAD activity increase (**Figures 1O–R**). TAZ protein expression was not increased by TNF treatment in YAP^−/−^ cells, thus showing that YAP is necessary for TAZ increase upon TNF treatment (**Figure 1N**). Altogether, these results demonstrated that TNF administration increased YAP nuclear localization and YAP-mediated TEAD transcriptional activity.

### TNF Activates YAP Through Rho Family of GTPases

**Figure 2:**
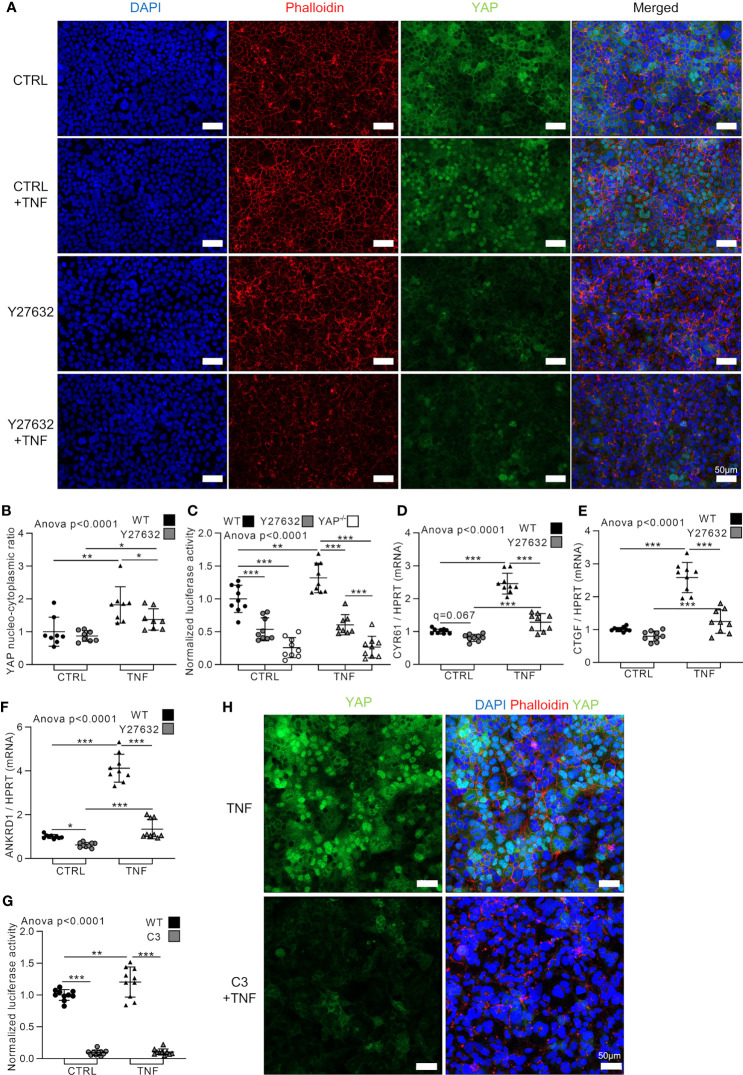
TNF activates YAP through Rho family of GTPases. HEK293 were cultured as described in **Figure 1**. Cells were pre-treated with Y27632 at 10 µM or C3 exoenzyme cell permeable at 2 µg/ml for 2 h before adding TNF for 48 h [24 h in **(C)** and **(G)**]. **(A)** Representative airyscan confocal z-stack max intensity images of YAP (immunofluorescence (IF) technique, green), phalloidin (actin, red), DAPI (nucleus, blue), and merged images. **(B)** Corresponding IF quantification of YAP nucleo-cytoplasmic ratio. **(C)** and **(G)** Luciferase reporter assay of TEAD transcription factor activity. **(D–F)** RT-qPCR results for CYR61 **(D)**, CTGF **(E)** and ANKRD1 **(F)**, results were normalized to HPRT expression. **(H)** Representative airyscan confocal z-stack max intensity images of YAP [immunofluorescence (IF) technique, green] alone or with phalloidin (actin, red) and DAPI (nucleus, blue). For confocal images, luminosity and contrast were enhanced identically for each image for clarity purpose. Results are representative of three independent experiments with 2 to 3 biological replicates for each experiment (n = 6 to 9 per group) with T-test or one-way ANOVA test and FDR corrected for multiple comparisons *post hoc* tests performed between conditions: *p < 0.05; **p < 0.01; ***p < 0.001. Data are expressed as fold change vs. control and presented as individual values with mean ± SD.

At cellular level, TNF induced profound changes in the actin network and increased the formation of F-actin resembling actin stress fibers. Indeed, the actin network in TNF treated cells appeared more tense and highly organized (**Figures 1A** and **2A**). Mechanotransduction events through Rho family of GTPases activity controls YAP nuclear translocation (7). To investigate if Rho family of GTPases are involved in YAP activation in response to TNF, HEK293 cells were treated with Y27632, a selective inhibitor of Rho-associated coiled-coil containing protein kinase (ROCK) activity downstream of Rho family of GTPases (41). As expected, Y27632 blunted F-actin organization, but did not affect YAP nucleo-cytoplasmic ratio (which was already low in our experimental conditions) compared to control cells (**Figures 2A, B**). However, Y27632 decreased YAP/TEAD activity (YAP^−/−^ cells were used as negative control) and slightly decreased YAP target genes CYR61, CTGF, and ANKRD1 in basal conditions (**Figures 2C–F**). Y27632 effects on YAP was less important than YAP KO which almost completely blunted TEAD activity (**Figure 2C**). As previously shown, TNF increased YAP nucleo-cytoplasmic ratio, YAP/TEAD activity, and YAP target genes expression (**Figures 2A–F**). However, in Y27632 treated cells, TNF slightly increased YAP nuclear translocation and YAP target genes expression but did not increase YAP/TEAD activity (**Figures 2A–F**). This YAP activation in Y27632 and TNF treated cells remained much lower than TNF treatment alone showing that TNF activated YAP mainly through Rho GTPases activity (**Figures 2A–F**). To confirm these results, a highly specific and potent Rho family GTPase (RhoA, RhoB and RhoC) inhibitor, C3 exoenzyme, was used (7). C3 administration strongly decreased TEAD activity in control and after TNF stimulation (**Figure 2G**). This was associated with a complete absence of YAP nuclear localization in C3 and TNF treated cells compared to TNF alone (**Figure 2H**). These results demonstrated that TNF effect on YAP transcriptional activity was mediated by Rho family GTases.

### YAP is Involved in the Gene Expression Profile Induced by TNF

**Figure 3:**
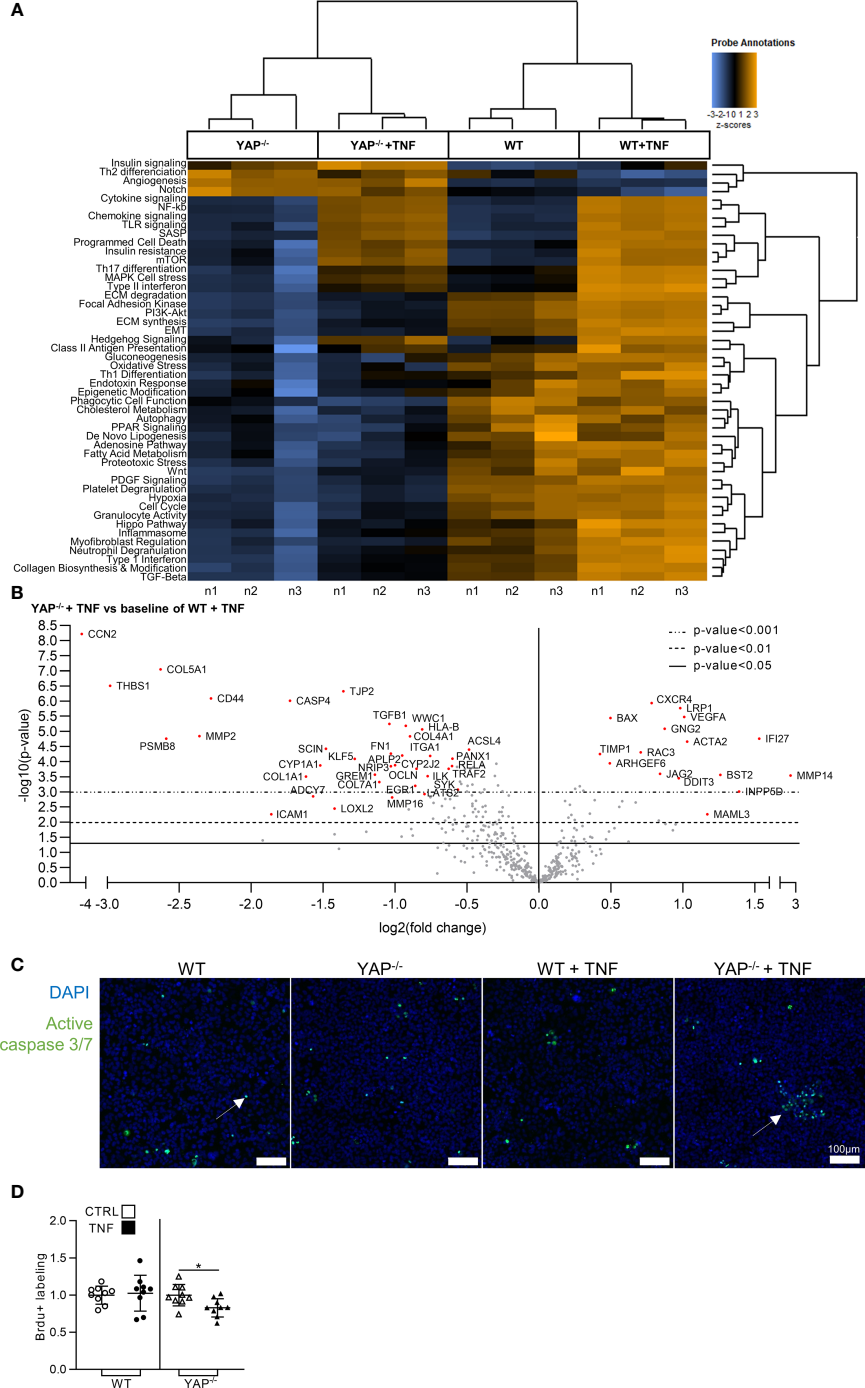
YAP modulates the expression of fibro-inflammatory pathways and promotes cell survival under TNF administration. HEK293 cells were cultured and treated with TNF as described in **Figure 1**. **(A)** Heat map of nanostring fibrosis panel pathways; pathways are listed to the left, the most upregulated pathways are depicted in orange, and the most downregulated pathways in blue; each column corresponded to one sample (n = 3/group). **(B)** Volcano plot representation for differential genes expression in YAP^−/−^ + TNF group versus the baseline of WT + TNF group; depicted genes were the most differentially expressed with the combination of a low p-value and a high fold change. **(C)** Representative live confocal images of active caspase 3/7 labeling (green) and DAPI (nucleus, blue). **(D)** BrdU assay results; results are representative of three independent experiments with 3 biological replicates for each experiment (n = 9 per group) and are represented as single values with mean ± SD and expressed as fold change vs WT control cells or YAP^−/−^ control cells; comparisons were only performed between CTRL and TNF treated cells, t-test *p < 0.05. For caspase labeling, images are representative of 3 independent experiments.

Since TNF activated YAP transcriptional activity, we explored the consequences of YAP KO on the gene expression profile induced by TNF treatment. Nanostring fibrosis panel, allowing the study of the expression of 770 genes, was used on YAP^−/−^ or WT HEK293 cells treated or not with TNF. The choice of the fibrosis panel was made because it included genes classically described to be responsive to TNF. The unsupervised analysis of pathways scores correctly attributed each sample to their respective groups (**Figure 3A**). As expected, TNF treatment in WT cells was effective to enhance gene expression related to pro-inflammatory (such as cytokine/chemokine and NF-κB signaling), and pro-fibrotic pathways (such as extracellular matrix (ECM) synthesis/degradation and TGF-β signaling) (**Figure 3A**). In contrast, YAP^−/−^ cells already displayed downregulation in several pathways compared to WT cells and responded weakly to TNF with moderate increase in inflammatory and no changes in fibrotic pathways (**Figure 3A**). Thus, most of differentially expressed genes were downregulated in YAP^−/−^ TNF treated cells compared to WT TNF treated cells (**Figure 3B**). As representative examples, among the top downregulated genes in YAP^−/−^ TNF treated cells compared to WT TNF treated cells, we found caspase 4 (CASP4), which is involved in inflammasome, and TGFB1 important for pro-fibrotic processes (**Figure 3B**). In the few upregulated genes in TNF treated YAP^−/−^ cells compared to TNF treated WT cells, BAX expression (a pro-apoptotic molecule) was increased, possibly suggesting a higher mortality of YAP^−/−^ TNF treated cells (**Figure 3B**). Interestingly, cell cycle pathway was also reduced in YAP^−/−^ cells with or without TNF treatment (**Figure 3A**).

### YAP Orients Cell Response to TNF Toward Survival and Promotes NF-κB Pathway Genes Expression

**Figure 4:**
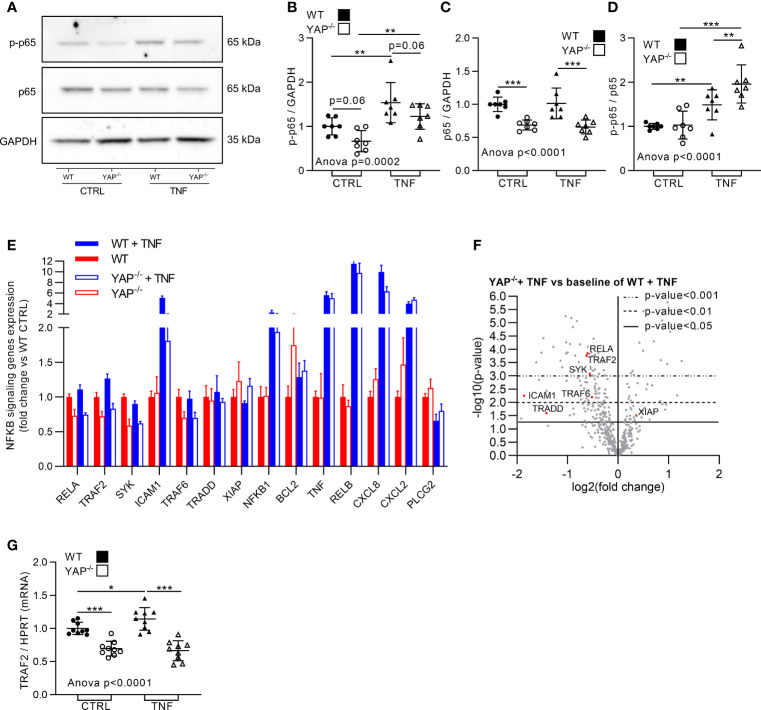
YAP modulates TNF effect on NF-κB pathway. HEK293 cells were cultured and treated with TNF as described in **Figure 1**. **(A–D)** Representative western blot of NF-κB p65 phosphorylation on serin 536 (active form, p-NF-κB), NF-κB p65 and GAPDH for WT and YAP^−/−^ control or TNF treated cells **(A)** with their respective quantifications as indicated **(B–D)**. **(E)** Nanostring fibrosis panel histogram for NF-κB pathway in the four groups; depicted genes were selected if at least one comparison between 2 groups gives a p-value <0.05 and must be related to NF-κB signaling. **(F)** Volcano plot representation for differential genes expression in YAP^−/−^ + TNF group versus the baseline of WT + TNF group; depicted genes are significantly differentially expressed and related to NF-κB pathway. **(G)** RT-qPCR quantification of TRAF2 expression normalized to HPRT expression. For WB and RT-qPCR, results are representative of three independent experiments with 2 to 3 biological replicates for each experiment (n = 7 to 9 per group) with T-test or one-way ANOVA test and FDR corrected for multiple comparisons *post hoc* tests performed between conditions: *p < 0.05; **p < 0.01; ***p < 0.001. Data are expressed as fold change vs. control and presented as individual values with mean ± SD **(B–D, G)** or presented as histogram with mean + SD **(E)**.

TNF could induce cell survival or apoptosis depending on the context (16). Our Nanostring results indicated differences in pro-survival pathways in the four groups. Thus, we hypothesized that YAP could be involved in the pro-survival response to TNF. Consequently, cell survival was assessed in the four groups. Active caspase 3/7 labeling (highlighting apoptotic cells) was similar in both unstimulated HEK293 (WT and YAP^−/−^), TNF had no impact on caspase 3/7 positive cells in WT HEK293 cells, but increased the number of apoptotic cells in YAP^−/−^ cells (**Figure 3C** **)**. Similarly, Brdu assay revealed that TNF had no impact on WT HEK293 cell proliferation, but reduced the proliferation of YAP^−/−^ cells (**Figure 3D**). So, YAP acted as a pro-survival factor following TNF treatment. To better understand how these phenotypic differences were regulated at molecular level, we investigated NF-κB pathway which is describe responsible to pro-survival cell phenotype under TNF treatment (16). In western blot, the phospho(p)-NF-κB (p65 subunit, phosphorylation on serine 536, active form involved in survival response) trended to be reduced in YAP^−/−^ cells in basal conditions, while TNF increased it to a lower amount in YAP^−/−^ cells than in WT cells (**Figures 4A, B**). The total amount of NF-κB was strongly reduced in YAP^−/−^ cells with or without TNF, thus consistent with a reduced expression of RELA [p65 subunit of NF-κB highlighted with Nanostring (**Figures 4A–F**)]. However, the p-NF-κB/total NF-κB ratio was higher in TNF treated YAP^−/−^ cells compared to untreated YAP^−/−^ cells and WT TNF treated cells highlighting a higher phosphorylation of the remaining NF-κB total protein in YAP^−/−^ cells treated with TNF (**Figures 4A, D** **)**. Furthermore, several other genes of the NF-κB pathways were downregulated in YAP^−/−^ cells (**Figures 4E, F**). Indeed, TRAF2, TRAF6, SYK, and TRADD were already downregulated compared to WT cells and were not increased by TNF treatment in YAP^−/−^ cells oppositely to WT cells (**Figures 4E, F**). Furthermore, ICAM1 expression was strongly increased by TNF treatment in WT cells, whereas this increase was much lower in YAP^−/−^ TNF treated cells (**Figures 4E, F**). However, several other NF-κB pathway related genes were increased by TNF in both WT and YAP^−/−^ cells such as RELB and NFKB1 (**Figures 4E, F**). Interestingly, TRADD is a major component of the TNF receptor and also TRAF2 and TRAF6 are critical to promote TNF signaling toward survival response and promote NF-κB signaling (16). We confirmed using RT-qPCR technique that TRAF2 expression was decreased in YAP^−/−^ cells and was not increased upon TNF treatment oppositely to WT cells (**Figure 4G**). Additionally, using chromatin immunoprecipitation followed by next-generation sequencing (ChIP-seq) data from previous report (42), YAP/TAZ/TEAD peaks were found at active enhancer sites of TRAF7, TRAF5, TRAF4, TRAF1, and TRAF2 genes (42). Altogether these results emphasized that TRAF2 is a YAP/TEAD target gene. To conclude, YAP was important for cell survival and NF-κB activity in response to TNF. YAP/TEAD role for TRAFs genes expression, being possibly responsible for the pro-survival effect of TNF.

### YAP Mediates TNF Effect on Pro-Inflammatory Gene Expression

**Figure 5:**
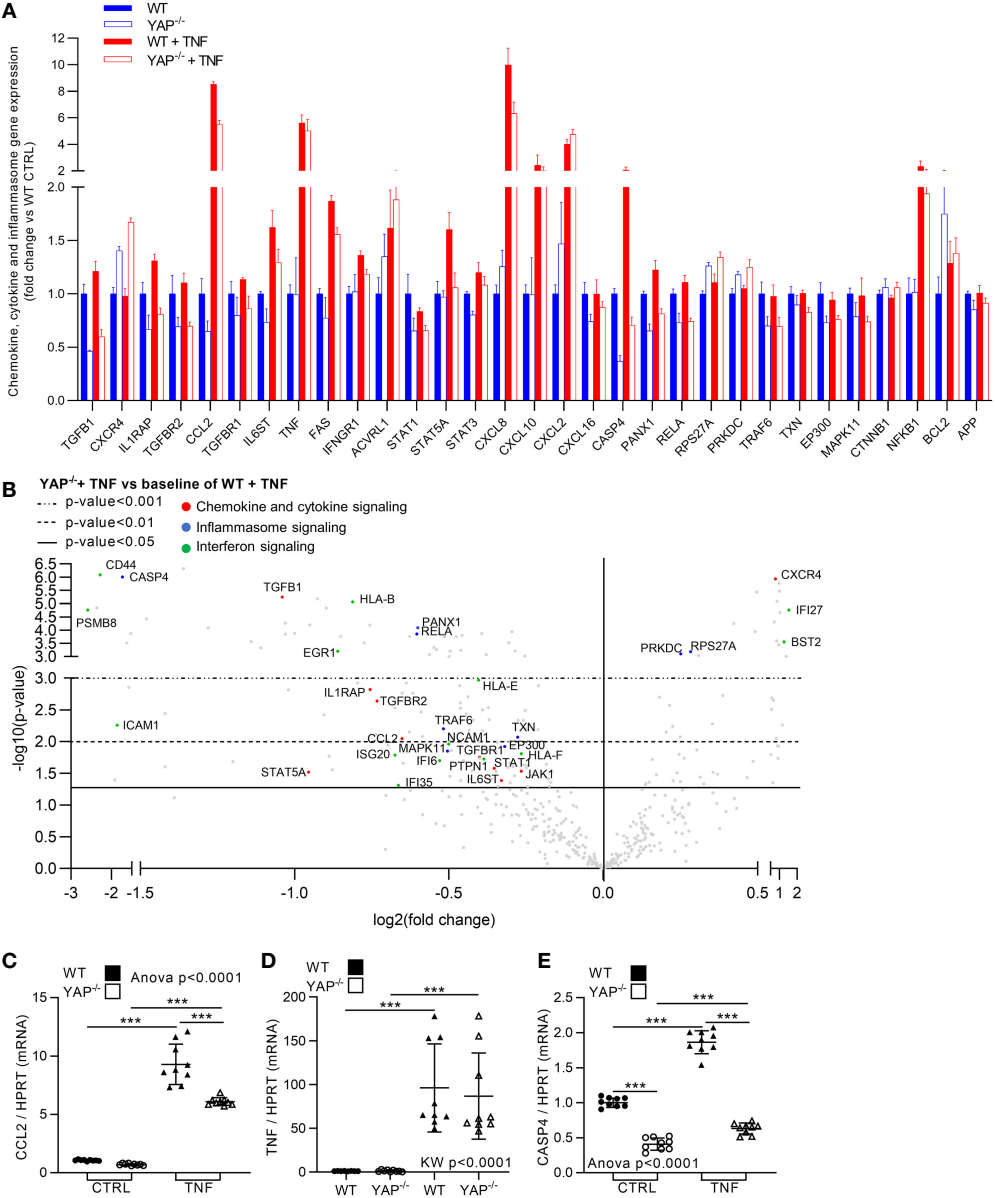
YAP modulates TNF effect on inflammatory genes expression. HEK293 cells were cultured and treated with TNF as described in **Figure 1**. **(A)** Nanostring fibrosis panel histogram for inflammatory pathways in the four groups; depicted genes were selected if at least one comparison between 2 groups gives a p-value <0.05 and must be related to chemokine, cytokine or inflammasome signaling. **(B)** Volcano plot representation for differential genes expression in YAP^−/−^ + TNF group versus the baseline of WT + TNF group; depicted genes are significantly differentially expressed and are related to chemokine/cytokine (red), inflammasome (blue) and interferon (green) pathways. **(C–E)** RT-qPCR quantification of CCL2, TNF and CASP4 expression normalized to HPRT expression. For RT-qPCR, results are representative of three independent experiments with 3 biological replicates for each experiment (n = 9 per group) with T-test or one-way ANOVA or Kruskal-Wallis (KW) test and FDR corrected for multiple comparisons *post hoc* tests performed between conditions: ***p <0.001. Data are expressed as fold change vs. control and presented as individual values with mean ± SD **(C–E)** or presented as histogram with mean + SD **(A)**.

Besides its role for cell survival, NF-κB is a master regulator of inflammatory response, thus NF-κB pathway reduction in YAP^−/−^ cells could be linked to reduce inflammatory response in YAP^−/−^ cells. In WT cells, TNF expectedly increased global inflammation. Indeed, in addition to NF-κB pathway increase, several other inflammatory pathways were increased by TNF treatment in WT cells such as cytokine/chemokine, inflammasome, and interferon signaling (**Figures 3A**, **5A, B** and **Figure S2**). Therefore, we chose to focus on these inflammatory pathways as they were strongly affected by TNF treatment in our results and are the most representative pathways of the pro-inflammatory response for *in vitro* models. In YAP^−/−^ control cells several genes belonging to these pathways where already altered compared to WT control cells, namely, TGFB1, IL1RAP, CCL2, IL6ST, STAT 1 and 3, CXCL16, CASP4, and PANX1. Furthermore, while TNF increased the expression of genes belonging to these pathways, such as IL1RAP, CCL2, IL6ST, FAS, STAT5A, CASP4, PANX1, RELA, CD44, PSMB8, EGR1, ICAM1, ISG20, JAK1, IFNGR1, and all HLA genes in WT cells, these genes were not affected or significantly less increased in YAP^−/−^ cells treated with TNF (**Figures 5A, B** and **Figure S2**). Thus, indicating that YAP was responsible, at least in part, for their expression upon TNF stimulation. On the other hand, some pro-inflammatory genes belonging to these pathways were increased equally between WT and YAP^−/−^ cells treated with TNF such as CXCL2 and TNF itself (**Figures 5A, B**). On the opposite, very few genes were increased more in YAP^−/−^ TNF treated cells compared to WT TNF treated cells such as CXCR4 (**Figures 5A, B**). RT-qPCR experiments confirmed Nanostring results for key genes of these pathways showing that YAP critically regulates the expression of CCL2 and CASP4 and had no effect on TNF expression itself (**Figures 5C–E**). Altogether these results demonstrate that YAP acts at transcriptional level to promote the expression of key pro-inflammatory genes. Thus, YAP transcriptional activity induction by TNF treatment mediated the expression of pro-inflammatory genes critical for the normal inflammatory response classically induced by TNF signaling.

### YAP Drives TNF Effect on Several Pro-Fibrotic Gene Expression

**Figure 6:**
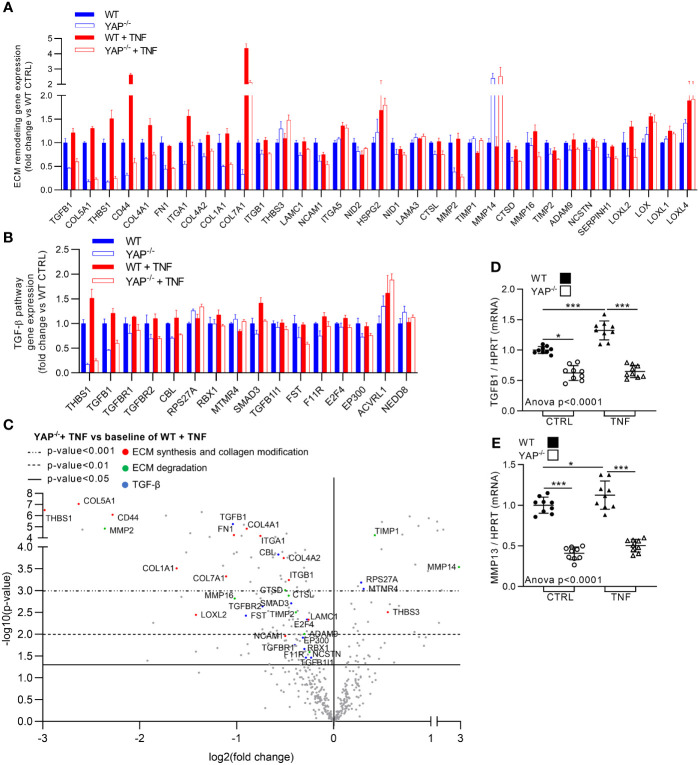
YAP drives TNF effect on pro-fibrotic genes expression. HEK293 cells were cultured and treated with TNF as described in **Figure 1**. **(A, B)** Nanostring fibrosis panel histograms for pro-fibrotic pathways in the four groups; depicted genes were selected if at least one comparison between 2 groups gives a p-value <0.05 and must be related to ECM remodeling (namely, ECM degradation, ECM synthesis and collagen biosynthesis and modification) **(A)** or TGF-β signaling **(B)**. **(C)** Volcano plot representation for differential genes expression in YAP^−/−^ + TNF group versus the baseline of WT + TNF group; depicted genes are significantly differentially expressed and are related to ECM synthesis and collagen biosynthesis and modification (red), ECM degradation (green) and TGF-β (blue) pathways. **(D, E)** RT-qPCR quantification of TGFB1 and MMP13 expression normalized to HPRT expression. For RT-qPCR, results are representative of three independent experiments with 3 biological replicates for each experiment (n = 9 per group) with T-test or one-way ANOVA test and FDR corrected for multiple comparisons *post hoc* tests performed between conditions: *p < 0.05; ***p < 0.001. Data are expressed as fold change vs. control and presented as individual values with mean ± SD **(D, E)** or presented as histogram with mean + SD **(A, B)**.

TNF is known to promote pro-fibrotic processes. Here, TNF expectedly increased the expression of genes related to pro-fibrotic pathways such ECM degradation and synthesis, collagen biosynthesis and modification (altogether referred as ECM remodeling in **Figure 6A**) and TGF-β pathway (**Figures 3A**, **6A–C**). In YAP^−/−^ untreated cells most of the genes belonging to these pathways were already altered compared to WT control cells with genes such FN1 and all collagens for ECM synthesis and collagen biosynthesis and modification, MMP2 for ECM degradation and TGFB1 for TGF-β signaling (**Figures 6A, B**). Furthermore, while TNF increased the expression of genes belonging to these pathways, such as TGFB1, THBS1, CD44, ITGA1, MMP16, ADAM9, LOXL2, RBX1, SMAD3, and all collagens in WT cells, these genes were not affected (or slightly increased) in YAP^−/−^ cells treated with TNF (**Figures 6A–C**). The few upregulated genes in YAP^−/−^ TNF treated cells compared to WT TNF treated cells belonging to these pathways seemed logical. Indeed, TIMP1 which is known to inhibit MMP activity (and which is logically decreased by TNF treatment in WT cells) was among these genes. Furthermore, MMP14 (which is responsible for MMP2 activation) and THBS3 increased expression could correspond to compensatory mechanisms (since MMP2 and THBS1 are strongly downregulated in YAP^−/−^ cells). RT-qPCR experiments confirmed Nanostring results for TGFB1 expression (**Figure 6D**). We also assessed MMP13 expression (which was not included in the Nanostring panel) by RT-qPCR showing that it was strongly downregulated in YAP^−/−^ control cells compared to WT control cells and did not increase upon TNF treatment oppositely to WT cells (**Figure 6E**). Altogether these results indicate that YAP^−/−^ cells have already in control conditions strong impairments in ECM remodeling and TGF-β pathways. Furthermore, these results demonstrated that YAP was mandatory for the induction of a pro-fibrotic gene expression profile under TNF stimulation.

### TNF Effect on Cytoskeleton and Focal Adhesion Kinase Pathway is Impaired in YAP^−/−^ Cells

**Figure 7:**
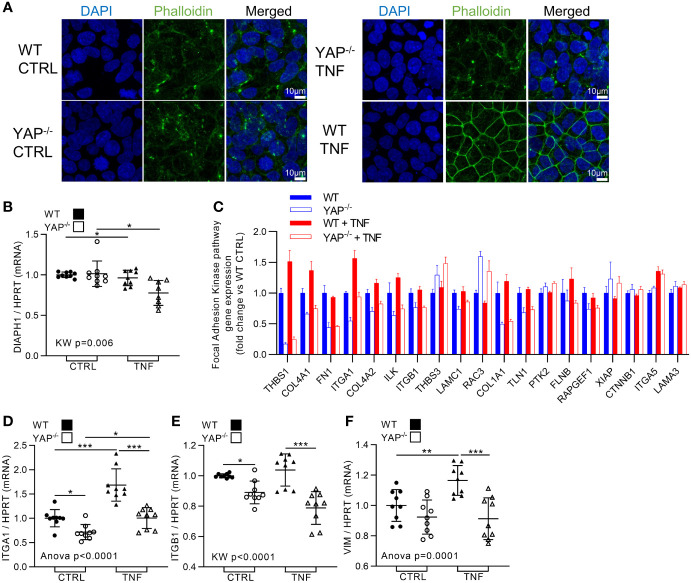
YAP drives TNF effect on cytoskeleton re-organization and focal adhesion kinase pathway. HEK293 cells were cultured and treated with TNF as described in **Figure 1**. **(A)** Representative airyscan confocal z-stack max intensity images of phalloidin (actin, green) and DAPI (nucleus, blue), and merged images (luminosity and contrast were enhanced identically for each image for clarity purpose). **(B)** RT-qPCR quantification of DIAPH1 expression normalized to HPRT expression. **(C)** Nanostring fibrosis panel histogram for focal adhesion kinase pathway in the four groups; depicted genes were selected if at least one comparison between 2 groups gives a p-value <0.05 and must be related to focal adhesion kinase signaling. **(D, E)** RT-qPCR quantification of ITGA1, ITGB1, and VIM expression normalized to HPRT expression. Confocal images are representative of three independent experiments. For RT-qPCR, results are representative of three independent experiments with 3 biological replicates for each experiment (n = 9 per group) with T-test or one-way ANOVA test and FDR corrected for multiple comparisons *post hoc* tests was performed between conditions: *p < 0.05; **p < 0.01; ***p < 0.001. Data are expressed as fold change vs. control and presented as individual values with mean ± SD **(B, D–F)** or presented as histogram with mean + SD **(C)**.

TNF is known to promote the migration and invasive abilities of cancer cells (18). This process, which is partly linked to ECM degradation (already presented in **Figure 6**), is also allowed by actin cytoskeleton remodeling and focal adhesion kinase (FAK) activity to help for cell motility and migration. Strikingly, YAP^−/−^ HEK293 were in the total incapacity to reorganize actin cytoskeleton upon TNF treatment contrarily to WT cells (**Figure 7A**), suggesting important defects in the control of actin organization in those cells. YAP transcriptional activity has been shown to control the formation of focal adhesion complexes and could also modify actin cytoskeleton dynamic by regulating the expression of MLC2 and DIAPH1 important for actin tension (43, 44). Using Nanostring results and RT-qPCR, we found that TNF had no effect on MLC2 (low expression in HEK293 cells) and do not increase DIAPH1 expression (which was, unexpectedly, decreased by TNF treatment in both WT and YAP^−/−^ cells) (**Figure 7B**). These results suggest that the actin organization differences in our model are not related to transcriptional changes in those genes. However, Nanostring results confirmed that YAP^−/−^ HEK293 cells display a decrease in the expression of key genes of focal adhesion components, namely, ITGA1, ITGB1, TLN1, ILK, and RAPGEF1 compared to WT cells (**Figure 7C**). Furthermore, TNF increased the expression of ITGA1, ILK and FLNB in WT cells, but not in YAP^−/−^ cells (**Figure 7C**). These transcriptional profile differences indicate integrin defects and could therefore explain the absence of actin reorganization in YAP^−/−^ TNF treated cells oppositely to WT cells. We confirmed using RT-qPCR the same differences in ITGA1 and ITGB1 expression (**Figures 7D, E**). Other cytoskeleton filaments are involved in invasive cell abilities such as vimentin (VIM), which could be increased by TNF signaling (45). We detected using Nanostring results differences in VIM expression (data not shown), and we confirmed using RT-qPCR that TNF increased its expression while this was not the case in YAP^−/−^ cells (**Figure 7F**). Altogether, these results indicated that YAP controlled the increase of focal adhesion genes expression and the cytoskeleton reorganization after TNF administration, thus possibly helping for cell migration and invasive abilities.

## Discussion

In this paper, we aimed to better understand YAP/TAZ interplay with pro-inflammatory signaling at cellular level by using TNF as the inflammatory input to study YAP/TAZ response. After having shown that TNF increased YAP/TEAD transcriptional activity, we discovered that YAP was responsible for the increase of numerous genes in response to TNF, indicating that YAP is an important effector of TNF signaling *in vitro*.

We first identified a clear YAP translocation to the nucleus and YAP/TEAD transcriptional activity in HEK293 cells treated with TNF. Despite the clear role of Rho family of GTPases for YAP activity under TNF treatment, more molecular studies are needed to better understand the temporality on how TNF modulates Rho/ROCK signaling and YAP transcriptional activity. YAP transcriptional activity inhibition was found after few hours of TNF treatment in HEK293 cells and was not assessed further than 6 hours of treatment (35). Our results indicate, however, that YAP transcriptional activity changes were undetected until an important increase at 24 h of treatment which was maintained at 48 h. Even in the condition of high basal YAP transcriptional activity (low cell density) TNF treatment did not decrease YAP nucleo-cytoplasmic ratio (still assessed after 48 h of TNF administration). This timing difference is the main hypothesis for explaining the opposite regulation of YAP upon TNF administration in HEK293 cells. Thus, it could be possible that TNF has a transient inhibitory effect on YAP signaling after a short period of time that then had a positive impact at a long-term scale. At the methodological level, an increase in YAP transcriptional activity at late timing was not linked to higher cell mortality upon TNF administration (which could result in a reduced cell density and consequently a higher YAP activity) since TNF had no impact on cell proliferation. We also found that the Hippo pathway was inhibited (reflected by lower YAPS127 phosphorylation) and that AMOT expression was reduced under TNF treatment, possibly contributing to YAP nuclear translocation. These events could be linked with Rho family of GTPases signaling since this inhibition completely prevented YAP nuclear translocation. However, we did not determine who comes first in these events nor if both the reduction of Hippo pathway and AMOT expression were needed for YAP nuclear translocation under TNF treatment. In any case, such long-term and prolonged activation of YAP seems more in accordance with a situation of chronic inflammation rather than an acute response to inflammatory signals. Thus, also reflecting *in vivo* or *ex vivo* studies where YAP is often found transcriptionally active in tissue where chronic inflammation happens (13, 46).

The increase in YAP transcriptional activity induced by TNF is needed for several biological processes. Indeed, YAP KO blunted the pathologic effect of TNF, by reducing genes involved in inflammatory, pro-fibrotic, and invasive responses, and oriented cells toward apoptosis instead of survival. We demonstrated that YAP promoted survival upon TNF stimulation since YAP KO cells proliferate less and die more. These differences could be explained in part by the ability of YAP/TEAD to modulate TRAF proteins expression. These proteins, and especially TRAF2, have been identified to induce pro-survival and pro-inflammatory responses through NF-κB and MAPK signaling upon TNF administration. Importantly, TRAF2 downregulation was found sufficient to induce pro-apoptotic response to TNF (16). Thus, YAP transcriptional control on TRAF2 expression could be critical to orient TNF response toward survival and inflammation rather than apoptosis. Anti-TNF therapy are already used for several inflammatory diseases (47) and have been shown effective in some cancers (18). However, complete inhibition of TNF activity in these situations is also detrimental because of the consecutive altered immune response and the loss of TNF pro-apoptotic activity. A better strategy is instead to re-sensitize cells to TNF pro-apoptotic effect to clear more efficiently cancer cells or abnormal pro-inflammatory resident cells in inflammatory disorders, this strategy has been already proposed (16). Our results indicate that this effect could be achieved by blocking YAP transcriptional activity, thus reinforcing the idea to target YAP in inflammatory related disorders. Furthermore, YAP KO also reduced the expression of NF-κB members themselves, in addition with RelA expression reduction (observed with Nanostring and Western Blot). However, the role of YAP for NF-κB activity is also controversial in the literature. Our work is in line with the work of others showing that YAP transcriptional activity could increase NF-κB signaling (33, 34, 48–50). On the opposite, strong evidence highlighted that YAP could inhibit NF-κB activity by interacting with and blocking upstream NF-κB signaling members such as TAK1 and TRAF6 leading to NF-κB retention in the cytoplasm (35, 38). This last activity of YAP could be related to its subcellular localization. A possible hypothesize is that, in the cytoplasm YAP inhibits NF-κB pathway, whereas in the nucleus it increases the transcription of NF-κB genes. Thus, TNF could has a double function by acting as a cytoplasmic drain for YAP: TNF increases YAP destruction through hippo kinase removing it from the cytoplasm (35, 36), and induces its nuclear translocation through Rho-GTPase activity leading to nuclear translocation. These two mechanisms could also be sequentially activated in time. Interestingly, in our model, TNF treatment do not only promote YAP localization in the nucleus, but also reduced its cytoplasmic localization, which in this case, could have a double positive effect on NF-κB activity. However, to test this hypothesis further *in vitro* investigations are needed.

In our model, YAP KO also reduced the expression of pro-inflammatory cytokines which are highly increased by TNF treatment in WT cells. Thus, in addition to promote survival upon TNF treatment, YAP acts as a sustainer of the inflammatory response induced by TNF. YAP is also responsive to other pro-inflammatory mediators. For example, we showed that IL-17 enhanced YAP/TEAD activity. Others found that YAP is also responsive to IL-6 (13), IL-1β (37) and YAP/TEAD transcriptional activity could promote the expression of pro-inflammatory mediators such as CCL2 (37, 51) and IL-8 (9). Overall, these results indicate that YAP mediates a broad pro-inflammatory response induced by a wide variety of pro-inflammatory mediators, reinforcing again the idea to target YAP during inflammatory related disorders.

Chronic inflammation is an important promotor of fibrosis. On one hand, chronic inflammation induces tissue stiffening through ECM remodeling, which could in turn, lead to YAP activation by mechanotransduction (46). On the other hand, YAP was already known to promote the expression of pro-fibrotic genes and fibrosis (52). Here we demonstrate that YAP is mandatory for the control of several pro-fibrotic gene expression (including TGF-β and ECM remodeling) upon inflammatory stimulation by TNF. This indicates that YAP not only passively responds to inflammation after tissue stiffening, but YAP acts as a molecular link between inflammation and fibrosis. Thus, YAP inhibition in *in vivo* inflammatory models could reduce inflammation but also fibrotic process. ECM degradation is also required to facilitate cell migration and invasion. The important control of YAP on the expression of ECM degradation genes, on focal adhesion components and on the re-organization of the cytoskeleton following TNF administration, strongly suggests that YAP drives cell migrative and invasive abilities promoted by TNF signaling. In pathological situations involving TNF, blocking ECM remodeling and cell migrative abilities through YAP inhibition, could therefore be effective to reduce fibrosis and metastasis. To finish, this TNF-YAP axis could also be important in physiological response, especially for tissue healing. For example, YAP is activated by IL-6 to promote healing of the intestine (13). TNF is quickly released in wound tissues and helps for the early processes of healing (53). Thus, our results let us hypothesize that TNF, by increasing YAP activity in resident cells of wounded tissue, could help the healing by promoting ECM synthesis, cell survival, and migration. Overall, YAP response to TNF could help to explain several physiologic and pathologic effects of TNF.

To conclude, we report how TNF activates YAP *in vitro* and demonstrate a new and important function of YAP in the modulation of TNF response. YAP transcriptional activity promotes cell survival, enhances inflammatory response, and drives several pro-fibrotic gene expressions in response to TNF. Consequently, targeting YAP/TEAD transcriptional activity could be a promising new way to inhibit pathological effect induced by TNF, and probably by other cytokines, on cell phenotype in human inflammatory-associated diseases such as cancer and chronic inflammatory disorders.

## Materials and Methods

### Cell Culture and Experimental Design

HEK293 cells were cultivated in Dulbecco’s modified Eagle’s medium (DMEM, Sigma, St. Louis, MO, US) with 10% fetal bovine serum (FBS), 1% non-essential amino acid solution and 2% penicillin and streptomycin (PS). HEK293 YAP^−/−^ were generated using specific CRISPR cas-9 and homology direct repair plasmid targeting YAP sequence (Santa Cruz Biotechnology, Dallas, TX, US), CRISPR clones generation was done following manufacturer instructions, and validated by western blot and DNA sequencing. Plates were coated with fibronectin (1:100, Sigma) for 2 h at 37°C before seeding. HEK293 were plated at high cell density (100,000 cells/cm^2^) or low cell density (10,000 cells/cm^2^). Approximately 24 h after seeding, TNF was used between 2.5 and 50 ng/ml (except if specified) and IL-17 at 50 ng/ml (R&D system, Minneapolis, MN, US. TNF or IL-17 treatment was performed for 48 h (except for luciferase assay). Y27632 (Sigma) was used at 10 µM and was pre-incubated for 2 h before TNF addition. C3 exoenzyme cell permeable (cytoskeleton, Denver, CO, US) was used at 2 µg/ml and pre-incubated for 2 h before TNF addition.

### Immunofluorescence

This technique was done on HEK293 cells fixed with 4% PFA at RT for 20 min. Cells were rehydrated, permeabilized in 0.3% Triton X-100, then blocked in 1% BSA, 5% goat serum and 0.1% Triton solution for 60 min at RT, and probed with the primary antibody diluted in the blocking solution overnight at 4°C. The following primary antibody was used: YAP (63.7 sc-101199, Santa Cruz Biotechnology; 1:100). After washing, cells were incubated with secondary antibody, goat anti-mouse 488 (A11034, Thermo Fisher) for 75 min at RT, all diluted at 1:400. Cells were counterstained with DAPI alone (10 min at 37°C) or coupled with phalloidin (R415, Thermo Fisher; or ab176753, Abcam) for 1 h at 37°C. Isotypic controls were always performed using mouse IgG isotype control (31903, Thermo Fisher), diluted at the same concentration as the primary antibody.

### Image Acquisition and Quantification

Images were acquired using a confocal laser microscope (LSM) 800 airyscan (Zeiss, Oberkochen, Germany) equipped with Zen software. YAP quantification in HEK293 cells were performed using automatic image J macro developed to allow the quantification of mean cytoplasmic intensity, mean nuclear intensity and nucleo-cytoplasmic ratio by dividing the two precedent parameters. This quantification was done by analyzing 2 to 3 ×200 magnification images per well representing 500 to 1,000 cells per images.

### Luciferase Assay

HEK293 cells were plated at 35,000 cells/well into 96 well plate. Approximately 24 h after cells were transfected using Jet prime (Polyplus transfection, New York, NY, US). Approximately 1 µg of plasmids (0.5 µg of each plasmid) with 2 µl transfection reagent were used for a 100 µl final volume 5 µl of the mixture was added to cells cultivated in 100 µl final volume in 96 well plate for 24 h. HOP flash plasmid which consist of 8× wild type TEAD binding sites with minimal promoter plus luciferase reporter gene (luciferase firefly) was transfected with pRL-SVl40P (renilla luciferase) expressing vector. TNF or IL-17 was added 24 h after transfection for 30 min, 2 h, 6 h or 24 h. After cell lysis luminescence was quantify using Promega dual glow assay (Promega, Madison, WI, US). After blank subtraction, Firefly activity was then divided by renilla activity for normalization. HOP-flash was a gift from Barry Gumbiner (Addgene plasmid # 83467, Watertown, MA, US) and pRL-SVl40P was a gift from Ron Prywes (Addgene plasmid # 27163).

### Protein Extraction and Western Blot

Protein extraction was performed with Allprep RNA/protein kit (Qiagen Inc, Hilden, Germany). Proteins (10 µg) were denatured and separated for 20 min at 200 V before being transferred onto PVDF membranes (Thermo Fisher Scientific). The membrane was blocked and incubated with primary antibody overnight at 4°C. Then membrane was incubated with a horseradish peroxidase-conjugated secondary antibody (1:5,000; Thermo Fisher Scientific, 31460) for 1 h at room temperature. Immunoreactive protein bands were visualized with Clarity™ Western ECL Substrate (BioRad, Hercules, CA, US). Western blot (WB) was performed using the following primary antibodies purchased from Cell Signaling Technology (Cell Signaling Technology, Leiden, The Netherlands) diluted at 1:1,000: p-YAPS127 (#4911), YAP/TAZ (#8418), NF-κB p65 (#8242), phospho NF-κB p65 ser536 (#3031) and 1:5,000: GAPDH (#2118).

### RNA Extraction and RT-qPCR

RNA was extracted using a Allprep RNA/protein kit (Qiagen Inc.). Quality and quantity of RNA were assessed by an Experion RNA analysis (BioRad) and QuantIT RiboGreen RNA assay (Thermo Fisher Scientifc), respectively. Complementary DNA (cDNA) was synthesized using the iscript cDNA synthesis kit (Biorad). Quantitative RT polymerase chain reaction (PCR) was conducted on CFX96 RealTime System (BioRad) with LightCycler FastStart DNA Master plus SYBRgreen I (Roche Diagnostics, Basel, Switzerland). The results were normalized to the housekeeping gene expression hypoxanthine-guanine phosphoribosyltransferase (HPRT).

### RNA Nanostring Technology

RNA (50 ng) was used from WT or YAP^-/-^ HEK293 treated or not with TNF (n = 3 per groups). The fibrosis gene panel was used including 770 genes involved in fibrotic processes. All quality controls were performed following manufacturer instructions. Normalization was performed using five housekeeping genes that were not affected by the experimental conditions. Count detection limit was determined using threshold based on negative controls. Data analysis was performed using the nSolver™ package (version 3.0) and Advanced Analysis module (version 1.0.36). Differential expression and pathway analysis were performed using the nSolver advance analysis module according to the guidance given by manufacturers. Genes with a p-value below 0.05 were considered as being significantly differentially expressed. To further confirmed these results, RT-qPCR were performed on some specific genes found differentially expressed with Nanostring panels, showing, for all genes tested, identical results.

### Brdu Assay

BrdU assay was performed using cell proliferation ELISA, BrdU colorimetric (Roche applied Science, Basel, Switzerland) following manufacturer instructions. Briefly, cells were cultured in 96 well plates with 100 µl of culture medium. BrdU labeling reagent was added to the wells 24 h before labeling. Medium was removed and cells were kept at 4°C for 24 h. Fixdenat solution was added for 30 min. Anti-BrdU antibody (1/100) was incubated for 90 min. After washing, substrate solution was added for 20 min before acquisition with spectrophotometer. All controls were performed accordingly to manufacturer instructions.

### Active Caspase3/7 Labeling

Cells were cultivated in 300 µl of normal culture medium in 8 wells µslide chambered coverslips (IBIDI, Gräfelfing, Germany). A stock solution containing Hoechst (1/1,000; Thermo Fisher Scientific) and CellEvent active caspase 3/7 green detection reagent (1/200; Thermo Fisher Scientific) was prepared. Before labeling, medium was partially removed from wells (200 µl), and 100 µl of the stock solution was gently added to the wells (Thus avoiding washing steps and preventing apoptotic cells detachment). After a 30 min incubation time, live imaging was performed on 3 fields per wells.

### Statistical Analysis

Data are presented as single values, with mean and standard deviation and are expressed as percentage of the mean of control values. Three independent experiments were always performed, with at least 2 biological replicates for each experiment. No data exclusion was performed except if samples were impossible to use due to low quality. Multiple comparisons were performed by one way ANOVA or Kruskal–Wallis test (according to normality), *post hoc* comparisons were corrected with the false discovery rate (FDR) method of Benjamini and Hochberg. Results were considered significantly different when p <0.05. All statistical analyses were performed on GraphPad Prism v9.2.0 software or nsolver advanced software (for Nanostring results).

## Data Availability Statement

The original nCounter Nansotring data presented in the study are publicly available. This data can be found here: https://www.ncbi.nlm.nih.gov/geo/query/acc.cgi?acc=GSE194136.

## Ethics Statement

Ethical review and approval was not required for the study on human participants in accordance with the local legislation and institutional requirements. Written informed consent for participation was not required for this study in accordance with the national legislation and the institutional requirements.

## Author Contributions

RC and HM designed the study. RC performed the experiments. ED contributed to RT-qPCR experiments. MC contributed to YAP immunofluorescence images quantification, WB and HEK293 low density experiments. MT contributed to RTqPCR and nanostring experiments. ML designed the mRNA primers. MN contributed to statistical analyses. RC analyzed the results. RC and HM wrote the paper. AG and LV made critical correction to the original manuscript. All authors listed have made a substantial, direct, and intellectual contribution to the work and approved it for publication.

## Funding

This work was supported by a Novartis DREAMER grant. The funder was not involved in the study design, collection, analysis, interpretation of data, the writing of this article or the decision to submit it for publication.

## Conflict of Interest

The authors declare that the research was conducted in the absence of any commercial or financial relationships that could be construed as a potential conflict of interest.

## Publisher’s Note

All claims expressed in this article are solely those of the authors and do not necessarily represent those of their affiliated organizations, or those of the publisher, the editors and the reviewers. Any product that may be evaluated in this article, or claim that may be made by its manufacturer, is not guaranteed or endorsed by the publisher.
