# Supplementary material for: YAP Transcriptional Activity Dictates Cell Response to TNF In Vitro
Source: Front Immunol. 2022 Mar 23;13:856247. doi: 10.3389/fimmu.2022.856247 (PMC8989468; doi:10.3389/fimmu.2022.856247)
Supplement: Supplementary file 1 [file DataSheet_1.docx]

Supplementary Material

##
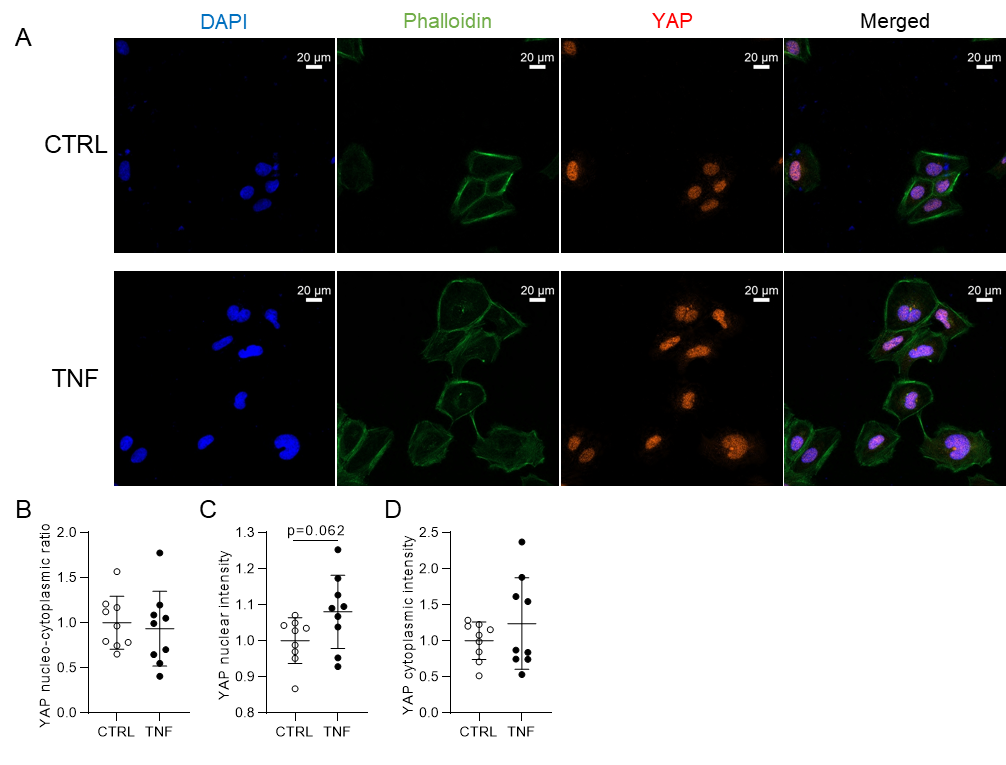
Supplementary Figures

**S. Figure 1. TNF has no effect on YAP localization at low cell density.** HEK293 were seeded at 10,000 cells/cm^2^ on fibronectin coated culture plate. Twenty-four hours after seeding cells were treated with TNF at 10ng/ml for 48 hours. A: Representative airyscan confocal z-stack max intensity images of YAP (immunofluorescence (IF) technique, red), phalloidin (actin, green), DAPI (nucleus, blue), and merged images (luminosity and contrast were enhanced identically for each image for clarity purpose). B-D: Corresponding IF quantification, with the nucleo-cytoplasmic ratio of YAP labelling (B), YAP mean nuclear intensity (C) and YAP mean cytoplasmic intensity (D). Results are representative of three independent experiments with n=3 per group for each experiment with T-test or ANOVA test and FDR corrected for multiple comparisons post hoc tests performed between conditions: * p<0.05; ** p<0.01; *** p<0.001. Data are expressed as fold change vs. control and presented as individual values with mean ± SD.


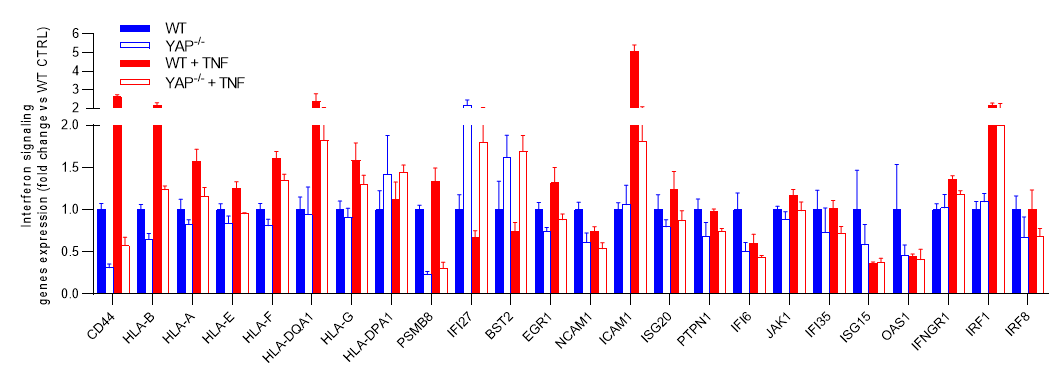


**S. Figure 2. YAP modulates TNF effect on interferon pathway gene expression.** HEK293 were seeded at 100,000 cells/cm^2^ on fibronectin coated culture plate. Twenty-four hours after seeding cells were treated with TNF at 10ng/ml for 48 hours. Nanostring fibrosis panel histogram representing mean ± SD, for interferon pathway in the four groups; depicted genes were selected if at least one comparison between 2 groups gives a p-value<0.05 and must be related to interferon signaling.
